# The sooner, the better: The economic impact of non‐pharmaceutical interventions during the early stage of the COVID‐19 pandemic

**DOI:** 10.1111/ecot.12284

**Published:** 2021-08-22

**Authors:** Asli Demirgüç‐Kunt, Michael Lokshin, Iván Torre

**Affiliations:** ^1^ The World Bank Office of the Chief Economist Europe and Central Asia Washington DC USA

**Keywords:** COVID‐19, economic impact, Europe and Central Asia, high‐frequency data, non‐pharmaceutical interventions, pandemic

## Abstract

This paper estimates the economic impact of the non‐pharmaceutical interventions (NPIs) implemented by countries in Europe and Central Asia during the initial stages of the COVID‐19 pandemic. The analysis relies on daily electricity consumption, nitrogen dioxide emission and mobility records to trace the economic disruptions caused by the pandemic and calibrate these measures to estimate the magnitude of the economic impact. To address the potential endogeneity in the introduction of NPIs, we instrument their stringency by the extent of a country's social ties to China. The results suggest that the NPIs led to a decline of about 10% in economic activity across the region. On average, countries that implemented non‐pharmaceutical interventions in the early stages of the pandemic appear to have better short‐term economic outcomes and lower cumulative mortality, compared with countries that imposed non‐pharmaceutical interventions during the later stages of the pandemic. Moreover, there is evidence that COVID‐19 mortality at the peak of the local outbreak has been lower in countries that acted earlier. In this sense, the results suggest that the sooner non‐pharmaceutical interventions are implemented, the better are the economic and health outcomes.

## 1 INTRODUCTION

One of the most contentious public policy debates since the beginning of the COVID‐19 pandemic has been about the economic and health consequences of non‐pharmaceutical interventions (NPIs). At the onset of the pandemic, when vaccines were not available and later when their availability was limited, NPIs appeared to be the only feasible instrument available to country governments to contain the spread of the disease. This paper contributes to the debate by quantifying the economic impact of NPIs and by showing how the timing and stringency of these interventions matter for their effect on the economy.

NPIs have a systemic impact on the economy. The closure of businesses and disruptions of the global value chains lead to direct loss of revenue, unprecedently high unemployment rates and sharp declines in personal incomes. However, if they are successful in containing the spread of the disease and thus improving the population's health, NPIs can limit the economic disruption associated with the disease itself, usually triggered by precautionary behaviour from consumers and producers. Which of these two effects prevail is a matter of empirical analysis, which we explore in this paper by illustrating the economic impact of the different types of NPIs implemented by countries in Europe and Central Asia during the initial stages of the COVID‐19 pandemic. We rely on high‐frequency proxies to trace the disruptions caused by the NPIs. These proxy measures are calibrated to provide an economic magnitude of the effects. To address the potential endogeneity in the introduction of stringent NPIs, we instrument their implementation by the degree of a country's social ties to China, the country where the first COVID‐19 cases were detected.

Our results show that national lockdowns – the most stringent type of NPI – led to a decline of about 10% in economic activity across Europe and Central Asia during March–April 2020. Countries that implemented a national lockdown once the disease was spreading extensively in their communities experienced a stronger decline in economic activity than those that implemented the same measure when there was limited community circulation of the virus. Similarly, for the first wave of the pandemic, there is suggestive evidence that COVID‐19 mortality at the peak of the local outbreak was lower in countries that acted earlier. The analysis also shows that the spread of the disease itself had a negative effect on economic activity that, at the peak of the outbreak, could rival that of the NPIs themselves. These results demonstrate that the limited negative economic impact of early NPIs can be attributed to their success in containing the disease and the disruption associated with it, as well as the fact that they were also less stringent in the restrictions they imposed.

An important caveat of our analysis is that it pertains to the first wave of the COVID‐19 pandemic during the spring of 2020 in Europe, when most countries’ governments enjoyed the political capital needed to implement tough restrictions on mobility and economic activity. As political capital was exhausted and ‘pandemic fatigue’ set in, repeated outbreaks of COVID‐19 that occurred in the fall of 2020 and the winter of 2021 became more challenging to deal with in the same way as the first wave earlier in 2020.

This paper is organized as follows. Section 2 presents a short literature review on the economic impact of NPIs and pandemics in general. Section 3 details the data used for the analysis and Section 4 illustrates the evolution of the pandemic and the implementation of NPIs in Europe and Central Asia. Section 5 presents further descriptive evidence of the impact on economic activity. Section 6 details the empirical analysis and presents the main results. Lastly, Section 7 addresses endogeneity concerns in the implementation of NPIs; Section 8 concludes indicating areas of further research.

## 2 PANDEMICS, NPIs AND ECONOMIC PERFORMANCE: A SHORT REVIEW OF THE LITERATURE

The COVID‐19 outbreak has triggered a large amount of scholarly work. Much of it builds upon the earlier analysis of past pandemics, notably of the 1918 ‘Spanish flu’. Brainerd and Siegler (2003) found that in the United States, the states most affected by the pandemic had stronger per capita income growth over the following decade and argued that this was due to increased labour productivity as measured by a higher capital–labour ratio. Garrett (2009) showed that higher mortality in 1918 was associated with higher wage growth at the city and state levels from 1914 to 1919. Almond (2006) found that a cohort in utero during the pandemic displayed worse education and health outcomes compared with other birth cohorts. Guimbeau et al. (2020) found a similar result for the cohorts born during the 1918 pandemic in São Paulo, Brazil.

Some of the key questions about the impact of the 1918 pandemic have been revisited recently.^1^ Barro et al. (2020) looked at the effect of influenza‐related mortality in 43 countries and found that the pandemic lowered real GDP between 6% and 8% in the typical country. Velde (2020) found that the recession triggered by the pandemic in the United States was sharp but short. Correia et al. (2020) demonstrated that cities in the United States that had higher mortality rates during the pandemic eventually had worse economic outcomes. They observed a similar negative impact on economic performance at the state level and showed that cities that implemented NPIs earlier on and for a longer time saw lower mortality peaks and cumulative mortality. Moreover, speedier, stricter and longer NPIs were associated with better economic outcomes in the long run.

One of the major subjects of discussion in recent work has been the role played by supply and demand forces. On the one hand, a pandemic may generate a negative supply shock by making workers sick and lower their productivity – and there is anecdotal evidence that this was a major source of business disruption during the 1918 Flu.^2^ On the other hand, consumers may react to the pandemic by cutting down on any type of consumption that requires interpersonal contact (Wren‐Lewis, 2020). Similarly, bleak economic prospects may depress private investment. Both effects can be characterized as demand shocks. However, the NPIs can also be seen as shocks to both supply and demand, as they force workers to stay at home, thus suppressing the production and preventing the consumption of certain services. Guerrieri et al. (2020) showed that the supply shock of social distancing and lockdowns triggered by the pandemic might generate a drop in aggregate demand larger than the supply shock itself.

In this context, the economic effect associated with NPIs has caught particular attention in the public debate because, in contrast to the 1918 flu pandemic, governments have been implementing more drastic measures to contain the outbreak.^3^ These interventions can create a severe economic downturn (Gourinchas, 2020). Eichenbaum et al. (2020) modelled the interactions between economic decisions and epidemics and found that the best containment policy can save many lives but may induce a more severe recession. However, Acemoglu et al. (2020) showed that targeted policies combined with measures that reduce interactions between groups and increase testing and isolation of the infected can minimize both economic losses and deaths. Similarly, Aum et al. (2021) calibrated a model to the progression of the pandemic in Korea and the United Kingdom and found that aggressive testing and tracking policies can reduce both the economic and health costs of COVID‐19. In this sense, a lives‐livelihoods trade‐off in the short run may not exist if targeted policies are feasible.^4^

In sum, while theoretical work on the COVID‐19 pandemic is abundant, little is known about its economic consequences. Most importantly for the academic and policy debate, whether the NPIs reduce both mortality and improve economic outcomes just as they did in 1918, and how these interventions interact with the supply and demand shocks triggered by the pandemic remain to be seen.

## 3 MEASURES OF ECONOMIC ACTIVITY AND DATA

The use of non‐monetary measures as a proxy for economic growth has gained the attention of economists in recent years. Most economic activities require electricity. For many countries, electricity data are available with a daily lag and on a subregional level, providing an almost real‐time picture of economic changes. Cicala (2020) demonstrated that, in the short run, changes in electricity consumption track standard economic indicators almost perfectly. Morris and Zhang (2019) proposed using satellite readings of tropospheric NO_2_ (nitrogen dioxide) densities as a proxy measure for economic activity. NO_2_ is a by‐product of the combustion of fossil fuels and, therefore, directly indicative of economic activity.^5^ Because the mobility restrictions are one of the main channels through which NPIs affect economic activity, any measure of mobility can be used as a measure of NPI enforcement or effective stringency. Location data derived from smartphones have become a popular way to illustrate mobility patterns by urban planners and transportation specialists.^6^ In the context of the COVID‐19 outbreak, Fang et al. (2020) studied the impact that the lockdown of Wuhan and Hubei province had on the spread of the disease using mobility patterns derived from the use of the smartphone mapping app of Baidu, China's most popular search engine.

In our analysis, we use five datasets, the first two covering proxy measures of economic activity, and the remaining covering information on mobility, NPIs and the evolution of the pandemic:

1. Electricity consumption. Data are presented as the total daily consumption in megawatts and were obtained from ENTSO‐E and national grid operators. Data are available for 37 counties in Europe and Central Asia; the period covered is 1 January 2017 to 17 April 2020.
2. NO_2_ emissions (tropospheric vertical column densities, or VCD) were obtained from the Ozone Monitoring Instrument (OMI) on NASA’s Aura satellite. The data are presented at the daily frequency for the entire world in pixels of 0.25 degrees of longitude × 0.25 degrees of latitude. The mean NO_2_ VCD value is computed for all pixels corresponding to the surface of a country, and the 30‐day moving average is used as the main variable of interest; the period covered is 1 January 2018 to 17 April 2020.
3. Mobility trends data produced by Apple and derived from the requests for directions using Apple Maps are available for 33 countries in Europe and Central Asia. The trends data distinguish mobility by types – driving and walking. The period covered is 13 January 2020 to 21 April 2020.
4. Data on the implementation of NPIs come from the Oxford Government Response Tracker, World Bank Education Global Practice COVID‐19 dashboard and alternative news sources.
5. Data on daily infections and deaths from COVID‐19, by country, from Our World in Data. The period covered is 1 January 2020 to 27 April 2020.

## 4 THE EVOLUTION OF THE COVID‐19 PANDEMIC AND THE IMPLEMENTATION OF NPIs IN EUROPE

The first case of COVID‐19 in Europe was reported in France on 24 January 2020. For the following weeks, reported cases were few and related to travellers coming from East Asia. On 19 February, a case of a man who had no known travel to at‐risk countries was reported in the region of Lombardy in Northern Italy. On 22 February, the first death by COVID‐19 was reported in the neighbouring region of Veneto, signalling the presence of community circulation of the virus in Northern Italy. In the days that followed, the infection cases and deaths by COVID‐19 in Italy saw an exponential growth, and Spain witnessed a similar outbreak. The pandemic spread to Belgium and France, and later to the Netherlands, Switzerland and the United Kingdom.

**FIGURE 1:**
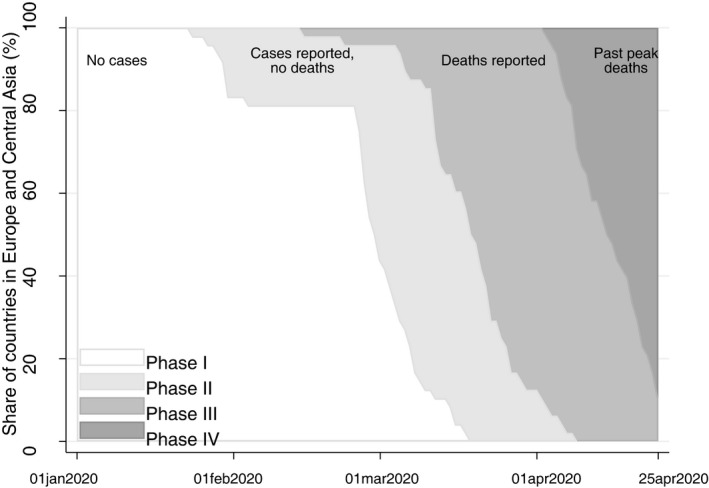
Evolution of COVID‐19 pandemic by phases *Note*: This graph plots the share of countries in Europe and Central Asia in each phase of the local COVID‐19 outbreak at each date between 1 January and 25 April 2020. Phase I corresponds to the period where no cases were reported in the country. Phase II corresponds to the period where cases of COVID‐19 were reported, but no deaths were caused by the disease. Phase III corresponds to the period where deaths by COVID‐19 are reported, and the daily figure is regularly increasing. Phase IV corresponds to the period where the peak of daily deaths by COVID‐19 has passed. The peak of daily deaths is determined as the highest 7‐day moving average of daily deaths.

We represent the evolution of the pandemic at the country level in four phases: in the first phase (I), no cases are reported in the country. In the second phase (II), infection cases are reported, but no deaths. In the third phase (III), deaths are reported, and the number of daily deaths increases until it peaks. The fourth phase (IV) starts after the peak in daily deaths is reached, and the daily number of deceased starts decreasing. Figure 1 plots the share of countries in the region in each phase by date. In February 2020, most countries were in phase I and only a handful were in phase II. By mid‐March, all countries were either in phase II or phase III. In early April, some countries started moving into phase IV, and by mid‐April, more than half of them had already passed the peak of daily deaths.

As mentioned before, in the absence of a vaccine, NPIs are the only tool available to limit the spread of COVID‐19. Around the end of January 2020, countries in Europe imposed restrictions on travel originating in East Asia; no wide‐ranging NPIs were implemented at that time. Only a few East Asian countries – the Republic of Korea; Taiwan, China; Vietnam; and Singapore managed to control the outbreaks with extensive testing and contact tracing. These countries, having been exposed to the SARS outbreak in 2002–2003 and MERS in 2015, had developed the capacity to ramp up testing and contact tracing by the time they detected the first cases of COVID‐19 (Bali et al., 2020). Western countries were not well prepared for the outbreak. To date, non‐communicable diseases represent most of the burden of disease in the West, and no tests for the new disease are available at scale (Institute for Health Metrics & Evaluation, 2017). For most non‐East Asian countries, the only available NPI that could contain the spread of COVID‐19 was a lockdown. Only when massive testing capacity was developed – in record speed in many cases – did governments start considering the possibility of softening the lockdown and moving to milder NPIs.

We focus on four types of NPIs adopted by the countries as the pandemic started to spread: (1) broad social distancing measures, as captured by the cancellation of public events and large gatherings; (2) the closure of schools; (3) the implementation of partial or targeted lockdowns; and 4) the implementation of full or general lockdowns. Appendix 1 details how the implementation date of each of these NPIs is defined and presents the adoption dates for each country in Table A1.

The first trigger for implementing NPIs appears to have been the detection of the community outbreak in Lombardy, Italy. After the Italian government established a ‘red zone’ forbidding entry and exit out of two clusters of towns in the regions of Lombardy and Veneto on 23 February, other countries started implementing social distancing measures, and a few decided to close schools. Only after the Italian government went for a full lockdown, first in Lombardy on 8 March and in the whole country on 10 March, the NPIs started being widely implemented in the region. Broad social distancing measures were in place in more than half of the European countries by 12 March, and half of the countries had closed schools by 13 March. Partial lockdowns were enforced in some countries, to be quickly replaced by full lockdowns. Half of the region was under complete lockdown by 21 March. By 9 April, all countries of Europe except Belarus, Sweden, Tajikistan, Turkey and Turkmenistan were in full lockdown.

Table 1 indicates the mean value of the epidemic peak – defined as the highest 7‐day moving average of daily deaths per million – for the countries that implemented different types of NPIs by the phase of the local outbreak. Countries that imposed a full lockdown before any deaths were reported had a mean peak of about 0.8 daily deaths per million. Countries that imposed a full lockdown after deaths were reported had a peak more than seven times higher at 6.29 daily deaths per million. A similar ratio of magnitude is found for the remaining types of NPIs. Note that this table does not intend to provide an estimate of the effectiveness of each type of NPI because countries have adopted more than one of them simultaneously, precluding the possibility of estimating such direct effects. However, it provides suggestive evidence that the epidemic curve appears to have been ‘flatter’ in those countries where NPIs were implemented in the earlier stages of the pandemic.

**TABLE 1:** Timing of NPI implementation and daily deaths at the epidemic peak

Type of NPI	Phases of the local outbreak at the time of implementation			
	I (no cases)	II (cases but no deaths)	III (deaths reported)	IV (past peak daily deaths)
	Mean (*median*) daily deaths per million at peak			
Ban of public events	1.19 *0.32*	2.75 *1.49*	11.22 *10.85*	–
School closure	0.41 *0.44*	1.16 *0.95*	11.75 *10.85*	–
Partial lockdown	–	1.05 *0.84*	6.22 *3.11*	–
Full lockdown	–	0.79 *0.85*	6.29 *2.81*	–

Note

This table presents the mean daily deaths per million (7‐day moving average) at the peak of the local COVID‐19 outbreak for the countries in each cell. The median value is indicated in italics. Values in this table are calculated with information from the 43 countries which had passed the peak by 25 April 2020. Social distancing is defined as the cancelling of public events and large gatherings. A partial lockdown only applies to a geographical region or a targeted set of activities.

## 5 THE IMPACT OF NPIs ON MOBILITY, ELECTRICITY CONSUMPTION AND NO_2_ EMISSIONS – A CURSORY LOOK AT THE DATA

We analyse the impact of NPIs on human activity throughout Europe and Central Asia. We start by presenting an example of descriptive results that show the impact of the COVID‐19 pandemic and the NPI responses on electricity consumption, NO_2_ emissions and mobility.^7^ We then provide a cross‐country illustration of the relationship between the implementation of NPIs and those variables, and in the next section, carry out a panel regression analysis to estimate the magnitudes.

The NPIs implemented in countries of Europe resulted in closure of businesses and a reduction in electricity‐intensive production. In the United States, grid operators have recently observed reduced weekday electricity demand relative to that expected for this time of the year and weather conditions (EIA, 2020). Similar patterns are observed for countries in Europe and Central Asia.

**FIGURE 2:**
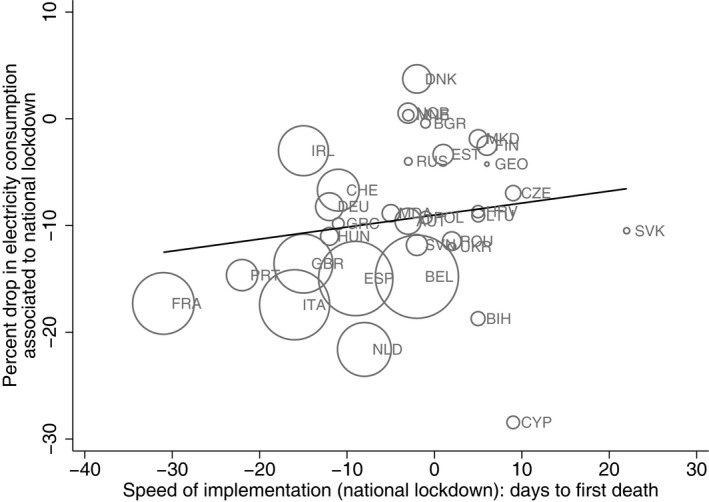
Electricity consumption by day of the year in Spain *Note*: The top left panel of this graph plots the daily consumption of electricity (grid electricity total load) for weekdays (weekends excluded) in the period between 1 January and 17 April 2019 (dotted line) and 2020 (solid line) in Spain. Values are normalized by electricity consumption on 1 January 2020. The top right panel plots the 30‐day running mean NO_2_ density (tropospheric column) between 1 January 1 and 11 April 2019 (dotted line) and 2020 (solid line). Values are normalized for each country by NO_2_ density on 1 January 2020. The bottom right panel shows these figures for Madrid. The bottom‐left panel shows the time spent driving and walking. Values are normalized by time driving and walking on 13 January 2020. Phase I identifies the period with no detected cases of COVID‐19; Phase II starts from the day when the first case is reported; Phase III begins at the date of the first death from the disease; Phase IV identifies the period after the peak of daily deaths in the country has been reached

The top left panel of Figure 2 shows changes in the consumption of electricity (grid electricity load) for Spain from 1 January to 17 April 2020 (solid line) and 2019 (dashed line). The shading in the figure darkens with the stages of the pandemic. The vertical lines represent the dates for the four types of NPIs. At the onset of the pandemic in February 2020, the use of electricity in Spain was already lower compared to the same period in 2019.^8^ The worsening pandemic and the introduction of NPIs in early March 2020 resulted in further decline. Spain consumed about 30% less electricity in April 2020 compared to April 2019. An uptick in electricity consumption in mid‐April most likely reflects the Government of Spain's decision to ease some lockdown restrictions.

NO_2_ emissions are closely correlated with traffic, construction activities, industry and coal‐fired power plants. The NPIs and especially national lockdowns imposed on the populations of most European countries resulted in a sharp drop in commuting and overall mobility and halt of many industrial activities. The reduction in NO_2_ emissions can be registered via satellite images and could potentially be used as a proxy for the economic impact of the pandemic (Davis, 2012).

The top right panel of Figure 2 shows changes in the levels of NO_2_. The NO_2_ levels in Spain were declining from January to April 2020, but until mid‐February, these levels were still higher than the levels in the corresponding days of 2019. After that point, the worsening pandemic and the introduction of NPIs were associated with a reduction in NO_2_ emission in Spain.

Lockdowns and other social distancing measures can have a direct impact on the personal mobility and operation of many businesses. Lockdowns restrict people's movements, allowing only a limited number of trips to grocery shops and pharmacies. Closure of most businesses and switch to home‐based work reduces travel to work. Real‐time data collected from mobile devices give a precise picture of the extent of the social distancing measures and the effectiveness of their enforcement.

The lower‐left panel of Figure 2 shows trends in the amount of time spent driving and walking by the day of the year in Spain. The saw‐like patterns of lines on the graph reflect changes in driving and walking over the days of the week. In phase I of the pandemic, personal mobility increases from Monday to Friday and declines over the weekend. The graph demonstrates a decline in driving and walking with the worsening pandemic, particularly after public events were banned on 10 March. Subsequent school closings and introduction of a full lockdown on 14 March reduced personal movements by almost 90% compared to the pre‐pandemic period. Very pronounced weekly mobility patterns almost disappeared after the lockdowns. These figures suggest that public compliance with social distancing measures was high, and the lockdown was strictly enforced.^9^

**FIGURE 3:**
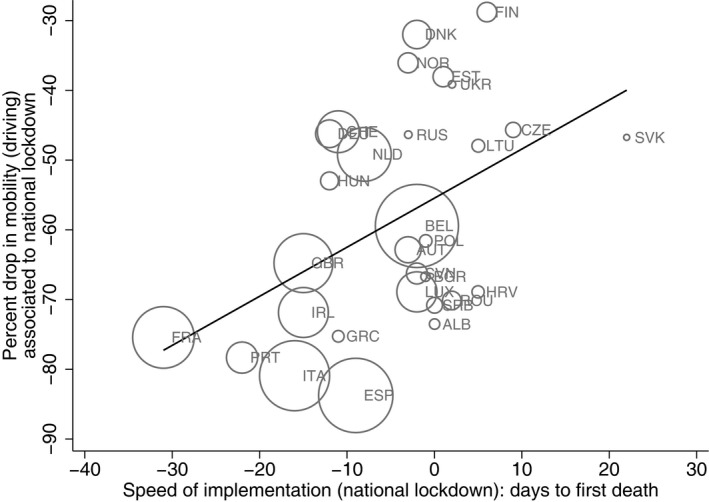
Change in electricity consumption and speed of implementation of national lockdown *Note*: This figure plots the relationship between the change in electricity consumption associated with a full, national lockdown (vertical axis) and the speed of implementation of the full lockdown (horizontal axis). The first variable is estimated from a country‐specific regression of daily electricity consumption (covering the period 2017–2020) on a series of days of the week, week of the year, holidays and temperature dummies, and a dummy variable indicating the implementation of a national lockdown following Cicala (2020). The coefficient of the national lockdown dummy variable is plotted on the vertical axis. The speed of implementation of the full lockdown is calculated as the number of days to the first reported death by COVID‐19 from the implementation date (i.e. date of first death – date of the lockdown). A negative value indicates that the full lockdown was implemented after the first death was reported; a positive value indicates that the lockdown was implemented before the first death was reported. The black line plots the linear fit between the change in electricity consumption and the speed of implementation. The size of the bubbles is proportional to the mortality rate per million inhabitants as of 25 April 2020

To take a cursory look at some of these relationships between the NPIs, the pandemic and economic activity, Figure 3 plots the change in electricity consumption associated with a full, national lockdown against the speed of implementation of a full lockdown, defined as the number of days between the implementation of the lockdown and the first reported death by COVID‐19. The size of the bubbles corresponds to the cumulative mortality rate per million inhabitants as of 25 April 2020. The per cent drop in electricity consumption is obtained from a country‐specific regression of daily electricity consumption over the period 2017–2020, on a series of days of the week, week of the year, holidays and temperature dummies, and a dummy variable indicating the implementation of a national lockdown following Cicala (2020). The coefficient of the lockdown dummy variable is plotted on the *y*‐axis. The figure indicates that countries that implemented a lockdown earlier on in the pandemic have seen lower overall drops in electricity consumption. As also hinted at in Table 1, earlier introduction of NPIs is also associated with lower mortality rates and lower cumulative mortality. Hence, the combined human and economic costs seem to have been lower for those countries that acted faster.

**FIGURE 4:**
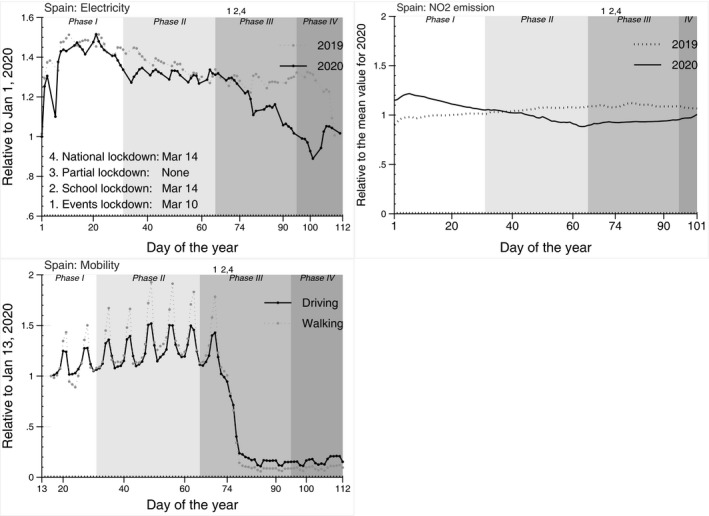
Change in mobility (driving) and speed of implementation of national lockdown *Note*: This figure plots the relationship between the change in mobility (driving) associated with full, national lockdown (vertical axis) and the speed of implementation of the full lockdown (horizontal axis). The first variable is estimated as the difference in the mean mobility index for driving during the implementation of the national lockdown and the mean mobility index for driving during the pre‐pandemic period (phase I: from 13 January 2020, to the day the first case was reported). The speed of implementation of the full lockdown is calculated as the number of days to the first reported death by COVID‐19 from the implementation date (i.e., date of first death – date of the lockdown). A negative value indicates that the full lockdown was implemented after the first death was reported; a positive value indicates that the lockdown was implemented before the first death was reported. The black line plots the linear fit between the change in mobility and the speed of implementation. The size of the bubbles is proportional to the mortality rate per million inhabitants as of 25 April 2020

This finding is also partially explained by the fact that countries that acted faster in implementing lockdowns were able to control the pandemic despite introducing less strict interventions. Using mobility as a proxy for the enforcement or effective strictness of the lockdown, Figure 4 shows that the speed of implementation is positively correlated with mobility: the reduction in ‘citizen’ mobility in response to the national lockdown is lower, the earlier the lockdowns are imposed. Using a measure of de jure stringency like the stringency index of government response (Hale et al., 2020), we observe a similar relationship. Hence speedier lockdowns also tend to be less stringent, though still associated with lower mortality. Acting earlier appears to allow governments to contain the pandemic more effectively and use less stringent measures, thus minimizing the economic costs. In the next section, we explore these associations more formally within a regression framework.

## 6 ESTIMATING THE EARLY ECONOMIC IMPACT OF NPIs

COVID‐19 pandemic impacted the economy of Europe through a number of channels. Directly, the pandemic reduced labour supply by affecting people's health and productivity. Uncertainties and fear of infection decreased consumer demand for goods and services, as people avoided public places and changed their consumption patterns. Responding to such a drop in demand and avoiding local outbreaks, businesses reduced worker density and thus labour demand: unemployment increased sharply. The closure of businesses and disruptions of global value chains led to direct revenue losses, high unemployment rates and sharp declines in personal incomes. Retail, transport, tourism and service industries, and small and medium‐size enterprises were affected the most.

The government‐imposed NPIs had a dual effect on the economy. On the one hand, lockdowns and social distancing restricted people's movement and reinforced the gravity of the pandemic situation; a large number of workers switched to home‐based work. School closures further reduced labour supply, especially among young parents. On the other hand, by containing the spread of the disease and improving the population's health, NPIs can limit the economic disruption associated with the pandemic. This section presents the empirical evidence on which of these two effects prevail.

Our baseline model relates the demand for electricity in a country, as a proxy of economic activity, to the implementation of NPIs, the evolution of the pandemic and a range of controls. Our model accounts for seasonality and weekly patterns in electricity consumption, as well as the changes in electricity demand during national holidays (following Cicala, 2020). We also control for differences in electricity consumption related to heating and cooling degrees.^10^ The basic specification we estimate is:

(1) $$Ln\left(Y_{i,t}\right)=\beta {\mathrm{NPI}}_{i,t}+\omega H_{i,t}+\theta {\mathrm{CH}}_{i,t}+\pi D_{t}+\gamma W_{t}+v_{i}+\epsilon_{i,t},$$

where *Y_c,t_* is the electricity consumption in country *i* on date *t*, *NPI_i,t_* is a vector of four dummies representing four types of NPIs, *H_i,t_* is equal to one if date *t* is a national holiday, *CH_i,t_* represents two variables for the heating and cooling days, *D_t_* are six dummies for days of the week,$W_{t}$ are the week of the year dummies, $v_{i}$is the country‐specific fixed effect and $\epsilon_{i}{,}_{t}$is an i.i.d. innovation term. β,ω,θ,πandγare the estimated parameters. In alternative specifications, we replace the electricity consumption by the level of NO_2_ emissions in the country, as another proxy activity measure. The level of NO_2_ emissions is a nosier variable, but it also likely to be impacted less by the pandemic itself. Each NPI dummy indicates a period when a particular NPI was the most stringent in place. From less to more stringent, the NPIs are a ban of public events, school closure, a partial or targeted lockdown and lastly, a full, national lockdown. For example, Germany imposed a ban on public events on 10 March, school closure on 16 March and a national lockdown on 22 March. For Germany, the dummy for the public event ban will have a value of one for the period from 10 March 10 until 16 March and zero afterwards; the school closure dummy switches on during the period from 16 March until 22 March and the national lockdown dummy activates on 22 March. The results are similar if each NPI dummy is included on its own in different specifications.

In specification (1), the coefficient β could be interpreted as estimates of the effects of the government‐mandated NPIs on both aggregate demand and supply.^11^ Social distancing measures reduce aggregate supply by forcing workers to stay at home and decrease aggregate demand by negatively affecting the consumption of services, particularly those that involve direct contact with customers or clients. The pandemic directly affects labour supply by reducing the number of workers because of sickness and lowering the productivity of sick workers. The fears and uncertainties associated with the progression of the pandemic resulted in a sharp increase in grocery spending and in a dramatic drop in expenditures on restaurants, retail, travel and public transportation that translates into reduced energy demand (Baker et al., 2020). To control for these effects, we expand specification (1) by adding health measures of pandemic progression. The new specification then becomes:

(2) $$Ln\left(Y_{i,t}\right)=\beta {\mathrm{NPI}}_{i,t}+\vartheta P_{i}{,}_{t}+\omega H_{i,t}+\theta {\mathrm{CH}}_{i,t}+\pi D_{t}+\gamma W_{t}+v_{i}+\epsilon_{i,t},$$

where *P_i,t_* is the daily number of deaths per million due to COVID‐19.^12^ Table 2 presents the descriptive statistics of the main variables used in the analysis.

**TABLE 2:** Descriptive statistics of main variables of interest

Variable	Observations	Mean	*SE*	Min	Max
Peak daily deaths per million (48 countries, whole sample)	48	3.70	6.03	0.02	29.19
Peak daily deaths per million (37 countries in electricity sample)	37	4.47	6.61	0.11	29.19
Daily deaths per million, 7‐day moving average (sample from February 1, 2020 to April 17, 2020)	2,561	0.91	2.87	0	29.19
Change in electricity consumption (phase 4 to predicted)	37	−0.087	0.084	−0.412	0.046
Change in NO_2_ emissions (2020 vs. 2019, same period)	48	−0.771	0.475	−0.633	2.417
Change in mobility, driving (phase 4 to phase 1)	33	−0.579	0.172	−0.837	−0.181
Mean stringency index for lockdown period (Oxford CGRT index)	26	80.2	9.69	51.4	99.3

Note

This table presents the mean, standard deviation, minimum and maximum for the main variables of interest in the empirical analysis. The unit of analysis for every variable in this table is the country, except daily deaths per million, for which it is country‐date. Electricity consumption, NO_2_ emissions and mobility are expressed as per cent changes relative to the corresponding reference periods. The mean stringency index is calculated as the average daily value of the stringency index during the days when a full lockdown was in place.

Any measure of pandemic progression is endogenous in specification (2) since unobserved factors could affect both the demand for electricity and the human impact of the pandemic. To address these concerns, we instrument the death rate in (2) with the daily predictions from a standard SIR epidemiological model that assumes an unmitigated spread of the disease (no NPIs implemented), and where the cross‐country variation comes only from pre‐pandemic characteristics like the demographic profile of the country, the number of ICU beds and an initial rate of contagion.^13^ There could also be concerns about the endogeneity of the timing of the NPIs. In particular, the speed with which policymakers act may itself be related to the characteristics and vulnerability of the economy to external shocks. We also address this issue below by instrumenting for the speed of interventions.

To assess the magnitude of the NPIs’ impact, it is necessary to assign a value to the elasticity between the proxy measures of economic activity and actual economic indicators. For electricity consumption, this elasticity is assumed to be 1 or close to 1 in the very short term (Cicala, 2020). For NO_2_ emissions – our alternative dependent variable – we estimate the elasticity to be between 0.32 and 1, with a midpoint at 0.66 (see Appendix 2).

Table 3 provides the fixed effect regression estimations of electricity consumption and NO_2_ emissions, for specifications (1) and (2). The last two pairs of columns present the IV estimations for both dependent variables. The estimation results of specification (1) without controls for the progression of the pandemic, shown in the first column of Table 3, demonstrate that, for 37 European countries, a ban on public events and a national lockdown leads, on average, to about a 11%–12% reduction in electricity consumption. Partial lockdowns and school closures have no significant impact on electricity consumption, most likely because, in many countries, the dates of implementation of the measures were close to when the national lockdown was imposed.^14^

**TABLE 3:** Fixed effect and instrumental variable fixed effect regression results of the response of electricity consumption and NO_2_ emission levels to the COVID‐19 pandemic and NPIs

	Log of electricity consumption				Log of NO_2_ emission level				Log of electricity consumption		Log of NO_2_ emission level	
	OLS				OLS				IV		IV	
	(1)		(2)		(3)		(4)		(5)		(6)	
	Coef.	*SE*	Coef.	*SE*	Coef.	*SE*	Coef.	*SE*	Coef.	*SE*	Coef.	*SE*
Non‐pharmaceutical interventions (NPI)												
National lockdown	−0.121***	0.012	−0.111***	0.012	−0.187***	0.034	−0.174***	0.036	−0.059***	0.028	−0.196***	0.036
Partial lockdown	0.037	0.023	−0.029	0.023	−0.118**	0.057	−0.114**	0.057	−0.012	0.030	−0.145**	0.060
School closure	−0.032	0.024	0.034	0.024	−0.114**	0.054	−0.112**	0.054	0.040*	0.024	−0.060	0.052
Ban on public events	−0.110**	0.007	−0.032	0.026	−0.244***	0.063	−0.243***	0.063	0.001	0.026	−0.313***	0.059
Pandemic progression indicators												
Daily deaths per million			−0.004**	0.002			−0.008	0.007	−0.027***	0.003	−0.004	0.008
First stage instruments												
Modelled death rate									0.336***	0.001	0.324***	0.002
*F* test									528.30		210.46	
Weak identification test									2,980		1,300	
No. of observations	43,535		43,535		8,487		8,487		43,535		8,487	
No. of countries	37		37		48		48		37		48	

Note

This table reports estimates from the following panel regression model: $Ln\left(Y_{i,t}\right)=\alpha +\beta_{\mathrm{NL}}{\mathrm{NL}}_{i,t}+\beta_{\mathrm{PL}}{\mathrm{PL}}_{i,t}+\beta_{\mathrm{SC}}{\mathrm{SC}}_{i,t}+\beta_{\mathrm{BP}}{\mathrm{BP}}_{i,t}+\vartheta P_{i}{,}_{t}+\omega H_{i,t}+\theta_{C}{{\mathrm{Cool}}_{i,t}+\theta_{H}Heat}_{i,t}+\pi D_{t}+\gamma W_{t}+{\mathrm{Year}}_{t}+v_{i}+\epsilon_{i,t}$

Where *Y_i,t_* is either daily electricity consumption (columns 1, 2 and 5) or daily 30‐day running mean of NO_2_ emissions (columns 3, 4 and 6) for country *i* on date *t*. *NL, PL, SC* and *BP* are dummy variables that take a value of 1 if a national lockdown (*NL*), a partial lockdown (*PL*), school closure (*SC*) or a ban of public events (*BP*) were in place in country *i* on date *t*. *BP* takes a value of zero if either *SC*, *PL* or *NL* takes a value of 1. *SC* takes a value of zero if either *PL* or *NL* takes a value of 1. *PL* takes a value of zero if *NL* takes a value of 1. *P_i,t_* is the 7‐day moving average of daily deaths by COVID‐19, expressed per million people. *H* is a dummy for the national holidays; *Cool* is a variable indicating the number of hours in day *t* where the average ambient temperature in country *i* was higher than 24ºC/75ºF. *Heat* is a variable indicating the number of hours in day *t* where the average ambient temperature in country *i* was lower than 18ºC/64ºF. *D* and *W* are the day of the week and week of the year dummies respectively; *Year* is a year dummy and *ν* is the country fixed effect. The coefficients for *H*, *Cool*, *Heat*, *D*, *W*, *Year* and *ν* are omitted from the table. Columns 1–4 estimate the panel model with ordinary least squares with fixed effects. In columns 5 and 6, *P_i,t_* is instrumented with the daily deaths by COVID‐19 estimated by an SIR epidemiological model that assumes unmitigated spread of the disease (no NPIs in place). The coefficients on the instrument in the first stage regression are also shown in columns 5 and 6. We report the first stage *F* statistic and the weak ID test, which is the Stock–Yogo weak identification test with critical values: 10% maximal IV size = 16.38; 15% = 8.96; 20% = 6.66; 25% = 5.9363. ^***^Indicates that the coefficient is significant at 1% level, ^**^at 5% level, ^*^at 10% level.

The second column of Table 3 shows the estimation of the specification with an added variable on the pandemic progression. The addition of daily deaths per million inhabitants does not have a significant effect on the lockdown coefficient but reduces the coefficient on the ban on public events significantly to 3%.^15^ An extra death per million inhabitants reduces electricity consumption by about 0.4%. The average peak of daily deaths in the sample is 4.5 deaths per million inhabitants, suggesting that the average country may have seen a decrease of close to 1.8% in electricity consumption associated with the pandemic at the pick of local outbreaks. Note that daily deaths per million vary in our sample from close to zero to almost 30, so the effect of the pandemic itself can potentially be large in an out‐of‐control outbreak.

The fifth column of Table 3 shows the results of the fixed effect regression, where the progression of the pandemic is instrumented by modelled predictions.^16^ Accounting for the endogeneity of the death rates further attenuates the effect of NPIs and increases the impact of the pandemic on electricity consumption to 2.7% for every daily death per million. Using these estimates, at the peak of the pandemic, even without any NPIs, the average country in the sample may have seen a decrease of 12% in electricity consumption. The impact of the pandemic is higher than the average 5.9% decrease in electricity consumption due to a full lockdown in the same specification, illustrating how both NPIs and the spread of the disease itself can be important in explaining the decrease in electricity consumption during the worst days of the pandemic.

When we use NO_2_ emissions as an alternative dependent variable in Table 3, we are able to extend the sample to include 48 countries in Europe and Central Asia. Because NO_2_ emissions tend to be noisy, we use a 30‐day running mean to smooth the data. However, NO_2_ levels are also less dependent on the behavioural response of the population to the progression of the pandemic, as they depend to a larger degree on industrial activity and transport. The results of the estimations using electricity consumption are confirmed by similar results when using NO_2_ emissions. Table 3 demonstrates that NPIs have a strong negative impact on the levels of NO_2_ emissions.^17^ Using the midpoint elasticities of economic activity with respect to NO_2_ emissions calculated (0.66), the −17% decrease in the levels of NO_2_ associated with a national lockdown would be equivalent to about a −11% decrease in aggregate output. This decline is similar to the estimates derived from the regressions on electricity consumption (where that elasticity is close to 1). As expected, the pandemic variable itself is not significant in these regressions.

Table 4 expands the baseline specification to explore the effect of the speed of NPI implementation and the stringency of NPIs on electricity consumption. The speed of NPI implementation is again defined as the number of days elapsed from the date when an NPI was implemented to the date of the first death from COVID‐19. That variable takes the value zero if an NPI is introduced on the day of the first death, with a faster implementation associated with more positive values and a slower implementation associated with negative ones. In the regressions, we focus on the speed of implementation of the national lockdown. We interact the national lockdown dummy with its corresponding speed variable. The stringency of the national lockdown is measured by two variables. The first variable represents the de jure stringency of the lockdown. It consists of the average value of the government response stringency index during the period when a full lockdown was in place. The index ranges from 0 (less stringent) to 100 (most stringent) and is based on the policy decisions taken by governments on several areas: workplace restrictions, mobility restrictions, school closure, and restrictions on gatherings and public events (Hale et al., 2020). This index is available only for 26 countries in our sample. An alternative measure of stringency, capturing the *effective* or de facto stringency of the lockdown, is the drop in mobility observed during the period when the lockdown was in place. Just as with the speed variable, in the regressions, we interact the stringency variables with the national lockdown dummy.

**TABLE 4:** Fixed effect regression results of the response of electricity consumption to the speed and stringency of NPIs

	Log of electricity consumption											
	(1)		(2)		(3)		(4)		(5)		(6)	
	Coef.	*SE*	Coef.	*SE*	Coef.	*SE*	Coef.	*SE*	Coef.	*SE*	Coef.	*SE*
Speed in implementation of national lockdown	−0.002^***^	0.001	−0.002^**^	0.001								
Stringency of the national lockdown					−0.001^**^	0.001	−0.001^*^	0.001				
Drop in mobility									−0.150^***^	0.010	−0.118^***^	0.010
National lockdown	−0.108^***^	0.013	−0.101^***^	0.013	−0.010	0.051	−0.009	0.051	0.037^***^	0.010	0.022^**^	0.010
Partial lockdown	−0.028	0.032	−0.017	0.032	−0.007	0.036	0.003	0.037	−0.036^***^	0.011	−0.034^***^	0.011
School closure	0.038	0.026	0.039	0.026	0.050	0.034	0.051	0.034	−0.004	0.009	−0.004	0.008
Ban on public events	−0.094^**^	0.034	−0.094^**^	0.034	−0.066^*^	0.036	−0.066^*^	0.036	0.011	0.008	0.007	0.007
Daily deaths per million			−0.003	0.002			−0.003	0.002			−0.005^***^	0.000
No. of observations	39,896		39,896		33,861		33,861		2,781		2,781	
No. of countries	34		34		29		29		27		27	

Note

This table reports estimates from the following panel regression model: $Ln\left(Y_{i,t}\right)=\alpha +\beta_{\mathrm{NL}}{\mathrm{NL}}_{i,t}+\beta_{\mathrm{NLD}}{\mathrm{NL}}_{i,t}\times {\mathrm{Speed}}_{i}+\beta_{\mathrm{NLS}}{\mathrm{NL}}_{i,t}\times {\mathrm{Stringency}}_{i}+\beta_{\mathrm{NLM}}{\mathrm{NL}}_{i,t}\times {Mobility\_drop}_{i}+\beta_{\mathrm{PL}}{\mathrm{PL}}_{i,t}+\beta_{\mathrm{SC}}{\mathrm{SC}}_{i,t}+\beta_{\mathrm{BP}}{\mathrm{BP}}_{i,t}+\vartheta P_{i}{,}_{t}+\omega H_{i,t}+\theta_{C}{{\mathrm{Cool}}_{i,t}+\theta_{H}Heat}_{i,t}+\pi D_{t}+\gamma W_{t}+{\mathrm{Year}}_{t}+v_{i}+\epsilon_{i,t}$

Where *Y_i,t_* is the daily electricity consumption for country *i* on date *t*. *NL, PL, SC* and *BP* are dummy variables that take a value of 1 if a national lockdown (*NL*), a partial lockdown (*PL*), school closure (*SC*) or a ban of public events (*BP*) were in place in country *i* on date *t*. *BP* takes a value of zero if either *SC*, *PL* or *NL* takes a value of 1. *SC* takes a value of zero if either *PL* or *NL* takes a value of 1. *PL* takes a value of zero if *NL* takes a value of 1. *Speed* indicates the number of days elapsed from the implementation of the national lockdown to the day when the first death by COVID‐19 was reported in the country. A negative number indicates that the lockdown was implemented late, after the first death was reported. *Stringency* indicates the mean value of the stringency index of government response during the period when the national lockdown was in place. The index ranges from 0 (least stringent) to 100 (most stringent). *Mobility_drop* indicates the drop in mobility associated with the national lockdown measured as the difference in average mobility (driving) during the lockdown with respect to 13 January 2020. The coefficients *β_NLD_*, *β_NLS_* and *β_NLM_* capture the partial correlation of the interaction between the national lockdown dummy *NL* and the *Speed*, *Stringency* and *Mobility_drop* variables respectively. Columns 1 and 2 include only the interaction with *speed*; columns 3 and 4 include only the interaction with *stringency*; columns 5 and 6 include only the interaction with *Mobility_drop*. *P_i,t_* is the 7‐day moving average of daily deaths by COVID‐19, expressed per million people. *H* is a dummy for the national holidays; *Cool* is a variable indicating the number of hours in day *t* where the average ambient temperature in country *i* was higher than 24ºC/75ºF. *Heat* is a variable indicating the number of hours in day *t* where the average ambient temperature in country *i* was lower than 18ºC/64ºF. *D* and *W* are the day of the week and week of the year dummies respectively; *Year* is a year dummy and *ν* is the country fixed effect. The coefficients for *H*, *Cool*, *Heat*, *D*, *W*, *Year* and *ν* are omitted from the table. The panel model is estimated with ordinary least squares with fixed effects. The results in the last two columns of the table are most likely to be biased due to the reverse causality problem. We show these only for illustration purposes. ^***^Indicates that the coefficient is significant at 1% level, ^**^at 5% level, ^*^at 10% level.

The first two columns in Table 4 show that countries that implemented the national lockdown earlier experienced a smaller decline in electricity consumption compared to countries that imposed national lockdowns on later stages of the pandemic. A full lockdown implemented on the day of the first death is associated with a decrease in electricity consumption of 11%. For each day of delay in imposing the full lockdown, the electricity consumption drops by an additional 0.2%. Implementing the lockdown 1 week before the first death results in about 1.5% higher electricity consumption. The average delay in implementing the full lockdown in the sample is about 3.3 days, suggesting the average country incurred close to a 1% decrease in electricity consumption due to the late implementation of the full lockdown. When the daily death rate is included in the regression (column 2), the results barely change, and the daily death rate variable itself is not statistically significant. This is not surprising given that in this specification, the speed variable is capturing part of the variability of the evolution of the pandemic: as seen in Table 1, earlier introduction of NPIs leads to lower peak daily death rates. In this sense, these results also suggest that speedy NPIs are economically less damaging, potentially because they limit the decline in activity associated with the spread of the disease.

Another reason speedy NPIs can limit the economic cost is when they are also less stringent. Simple cross‐country correlations suggest that an additional week of delay in implementing a national lockdown is associated with a 1.4% more de jure stringent lockdown and a 5% more de facto stringent lockdown. We analyse the impact of de jure stringency of NPI implementation in columns 3 and 4 of Table 4. The average stringency of the national lockdown in the sample of 26 countries is 80.2 (on a scale from 0 to 100), implying that the average country saw a decrease of 8% in electricity consumption associated with the full lockdown. The most stringent lockdown in the sample – with the stringency index of 99.3 – is associated with an almost 10% decrease in electricity consumption. The least stringent lockdown – with the stringency index of 51.6 – is associated with a 5% drop. Again, once de jure stringency is included in the analysis, the effect capturing the evolution of the pandemic – as measured by the daily deaths by COVID‐19 – becomes statistically not significant (column 4). This suggests that, just like for the speed of the implementation, the stringency of a lockdown already captures most of the meaningful variation associated with the spread of the disease.

Columns 5 and 6 of Table 4 use the average drop in mobility associated with the lockdown as a measure of de facto stringency. These results should be interpreted with caution because mobility itself may have a direct effect on electricity consumption beyond any variation induced by the lockdown. There is a possibility of reverse causality – drops in economic activity (electricity consumption) may themselves cause a drop in mobility. Therefore, the estimated coefficients may be biased. Nevertheless, the estimated coefficient of 15%, evaluated at the mean reduction in mobility of 58%, suggests a drop in electricity use close to 9%, which is consistent with the de jure stringency estimates. Overall, these results suggest that both de jure and de facto more stringent lockdowns are indeed more economically damaging.

Delayed lockdowns compound the effect of the pandemic: cross‐country correlations show that for a given level of de jure stringency, de facto stringency is higher the slower the implementation of the lockdown,^18^ which is also associated with a higher mortality rate. These results are mirrored by emerging studies on the optimal trajectories for the post‐lockdown economic recovery, which demonstrates that countries that adopted a gradual and staged reopening experienced stronger economic recovery compared with countries that rushed into lifting the restrictive measures (Demirgüç‐Kunt et al., 2020b).

## 7 ADDRESSING ENDOGENEITY OF THE SPEED OF NPI IMPLEMENTATION

An important issue in our analysis is the potential endogeneity of the speed of implementation of the NPIs. For example, the speed of implementation of the NPIs is likely to be correlated with the country's quality of governance. Hence, the better outcomes observed for countries that implemented the NPIs earlier can be due to the quality of their governance and not to the speed of the intervention. In other words, had these countries implemented the NPIs later, the economic and health outcomes would still be favourable because of the quality of their superior governance. We try to address this critique in two ways.

First, the observed relation between the quality of the governance and the impact of the pandemic appears to work in opposite directions. Countries that suffered the heaviest economic and human losses are Western European counties (such as Belgium, France, Italy, Spain and the United Kingdom) with good governance indicators with respect to the region's average.^19^ These countries also implemented the NPIs later compared to other countries. So, our claims that sooner interventions are more effective in saving lives and reducing the economic costs are only strengthened by these observations. If the losses from the delayed implementation of NPIs are high for countries with good governance, such losses would be even higher for countries with a lower quality of governance. In other words, if the quality of governance matters, our estimates provide a lower bound of the impact of the delayed implementation of the NPIs.

**FIGURE 5:**
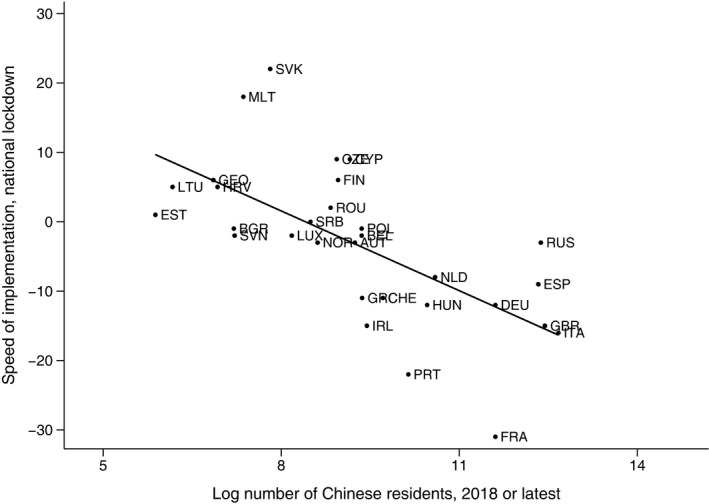
Speed of the national lockdown implementation versus log number of Chinese residents in a country *Note*: This figure plots the relationship between the number of Chinese residents in a country (horizontal axis) and the speed of implementation of the full lockdown (vertical axis). The speed of implementation of the full lockdown is calculated as the number of days to the first reported death by COVID‐19 from the implementation date (i.e., date of first death – date of the lockdown). A negative value indicates that the full lockdown was implemented after the first death was reported; a positive value indicates that the lockdown was implemented before the first death was reported. The straight line plots the linear fit between the speed of implementation and the log number of Chinese residents in a country

Second, we approach this critique formally and try to address the endogeneity of the speed of NPI implementation by using an instrumental variable approach. We use the number of residents from China as an instrument for the speed of NPI implementation.^20^ Given that the disease originated in China, it is probable that countries with stronger social ties to that country were the first ones to be exposed to the disease and therefore were only able to implement NPIs when the disease had already spread considerably; in other words, a late implementation. The number of Chinese residents serves as a proxy of the social ties of a country to China. Our exclusion restriction relies on the assumption that the number of migrants from China that accumulated over the years is not correlated with the current quality of governance in the country. Figure 5 shows the scatter plot of the speed of implementation against the number of migrants from China and illustrates that the countries with a lower number of migrants implemented the national lockdown faster than those with a higher number of migrants.

Table 5 shows the results of the baseline regressions (the same as columns 1 and 2 in Table 4) and the IV FE regressions where the speed of implementation of the national lockdown is instrumented by the number of Chinese residents in the country. The instrumented FE regression results are consistent with the baseline results. The IV FE regression in column 2 shows that a full lockdown implemented on the day of the first death is associated with a decrease in electricity consumption of about 9%. While the IV estimation attenuates the coefficient on the national lockdown dummy, the IV FE estimation increases the coefficient on the interaction term. Thus, each day of delay in imposing the full lockdown, the electricity consumption drops by an additional 0.5%, compared to 0.2% in the baseline regression. Implementing the lockdown 1 week before the first death results in about 3.5% higher electricity consumption. The average delay in implementing the full lockdown in the sample is about 3.3 days, suggesting the average country incurred close to a 2% decrease in electricity consumption due to the late implementation of the full lockdown. Including the daily death rates in the IV regression (column 4) has a trivial effect on the results; similar to the baseline specification, the daily death rate variable itself is not statistically significant.

**TABLE 5:** Fixed effect regression results of the response of electricity consumption to the speed and stringency of NPIs

	Baseline (1)		IV (2)		Baseline (3)		IV (4)	
	Coef.	*SE*	Coef.	*SE*	Coef.	*SE*	Coef.	*SE*
Speed in implementation of national lockdown	−0.002^***^	0.001	−0.005^***^	0.002	−0.002^*^	0.001	−0.005^***^	0.002
National lockdown	−0.108^***^	0.013	−0.088^***^	0.015	−0.101^***^	0.013	−0.089^***^	0.015
Partial lockdown	−0.028	0.032	−0.003	0.033	−0.017	0.032	−0.004	0.034
School closure	0.038	0.026	0.061^**^	0.031	0.039	0.026	0.061^**^	0.031
Ban on public events	−0.094^***^	0.034	−0.076^**^	0.036	−0.094^***^	0.034	−0.076^**^	0.036
Daily deaths per million					−0.003	0.002	0.000	0.003
Instrument			−1.070^***^	0.007			−0.973^***^	0.007
*F* test			2,593.99				2,543.34	
Weak identification test			23,995.63				16,638.49	
No. of observations	39,896		33,861		39,896		33,861	
No. of countries	34		29		34		29	

Note

This table reports estimates from the following panel regression model: $Ln\left(Y_{i,t}\right)=\alpha +\beta_{\mathrm{NL}}{\mathrm{NL}}_{i,t}+\beta_{\mathrm{NLD}}{\mathrm{NL}}_{i,t}\times {\mathrm{Speed}}_{i}+\beta_{\mathrm{PL}}{\mathrm{PL}}_{i,t}+\beta_{\mathrm{SC}}{\mathrm{SC}}_{i,t}+\beta_{\mathrm{BP}}{\mathrm{BP}}_{i,t}+\vartheta P_{i}{,}_{t}+\omega H_{i,t}+\theta_{C}{{\mathrm{Cool}}_{i,t}+\theta_{H}Heat}_{i,t}+\pi D_{t}+\gamma W_{t}+{\mathrm{Year}}_{t}+v_{i}+\epsilon_{i,t}$

Where *Y_i,t_* is the daily electricity consumption for country *i* on date *t*. *NL, PL, SC,* and *BP* are dummy variables that take a value of 1 if a national lockdown (*NL*), a partial lockdown (*PL*), school closure (*SC*) or a ban of public events (*BP*) were in place in country *i* on date *t*. *BP* takes a value of zero if either *SC*, *PL* or *NL* takes a value of 1. *SC* takes a value of zero if either *PL* or *NL* takes a value of 1. *PL* takes a value of zero if *NL* takes a value of 1. *Speed* indicates the number of days elapsed from the implementation of the national lockdown to the day when the first death by COVID‐19 was reported in the country. A negative number indicates that the lockdown was implemented late, after the first death was reported. The coefficient *β_NLD_* captures the partial correlation of the interaction between the national lockdown dummy *NL* and the *Speed* variable. The IV specifications (columns 2 and 4) instrument the speed of implementation with the log number of Chinese residents in the country. *P_i,t_* is the 7‐day moving average of daily deaths by COVID‐19, expressed per million people. *H* is a dummy for the national holidays; *Cool* is a variable indicating the number of hours in day *t* where the average ambient temperature in country *i* was higher than 24ºC/75ºF. *Heat* is a variable indicating the number of hours in day *t* where the average ambient temperature in country *i* was lower than 18ºC/64ºF. *D* and *W* are the day of the week and week of the year dummies respectively; *Year* is a year dummy and *ν* is the country fixed effect. The coefficients for *H*, *Cool*, *Heat*, *D*, *W*, *Year* and *ν* are omitted from the table. The panel model is estimated with ordinary least squares with fixed effects. For five countries in our sample, Bosnia and Herzegovina, Macedonia, Moldova, Montenegro and Ukraine, it was not possible to obtain the number of Chinese residents. ^***^Indicates that the coefficient is significant at 1% level, ^**^at 5% level, ^*^at 10% level.

## 8 CONCLUSION

The COVID‐19 pandemic has caused a huge economic and human cost since its outbreak in early 2020. Although there is ample theoretical work on the economic implications of the pandemic, empirical estimates of the size of the economic shocks triggered by the spread of the disease and associated NPIs are scarce. In this paper, we provide an illustration of the early economic impact by tracking the evolution of high‐frequency variables, which proxy economic activity. We show the effect of the pandemic on daily measurements of electricity consumption, NO_2_ emissions and mobility across Europe and Central Asia.

Proxy measures of economic activity allow us to investigate the economic impact of NPIs. It has been argued that these NPIs, while helpful in ‘flattening the curve’ of health outcomes, may come at high economic costs. Our results suggest that NPIs, and specifically national lockdowns, are associated with a decline in economic activity of around 10% across the region, whether we measure economic activity by electricity use or emissions data. Nevertheless, our empirical analysis shows that at least during the early stage of the pandemic, until the end of April 2020, countries that acted earlier – by implementing NPIs before the first deaths by COVID‐19 were reported – saw smaller drops in economic activity, in part because they were less stringent. Moreover, there is evidence that the COVID‐19 mortality at the peak of the local outbreak, as well as aggregate mortality, have been lower for countries that acted earlier. In this sense, our results suggest that the sooner NPIs are implemented, the better the economic and health outcomes.

The analysis in this paper also shows that the spread of the disease itself has an economic impact distinct from that of NPIs: at the peak of the outbreak, the drop in activity associated with the spread of the disease – be it by incapacitation of workers or by the precautionary reaction of consumers and investors – can be as strong as the shock triggered by lockdown measures. The smaller economic fallout of speedier interventions can also be explained by their effectiveness in containing the spread of the disease and, therefore, limiting the economic damage of the pandemic itself. These initial NPIs also provided a much‐needed breathing space for developing testing and contact tracing capacity in many countries, which were an essential component of pandemic response. Controlling for the impact of the pandemic itself and addressing endogeneity concerns regarding the pandemic and the speed of implementation of NPIs does not change these findings.

Our results are borne out of the patterns observed during the first wave of the COVID‐19 pandemic. Countries in Europe and Central Asia faced at least two more waves after it: one in the fall of 2020 and one in the following winter. National lockdowns were reimposed more than once, but a sense of ‘pandemic fatigue’ also started to diminish adherence to NPIs by the general population (Goldstein et al., 2021). Studying different waves of the pandemic, the process of different reopening strategies as well as reintroduction of NPIs will be important in this context. Also important is the role of trust and good governance in employing successful pandemic management strategies, whether they are vaccine deployment or continuation of mobility restrictions, in a prolonged fight with the virus variants. We leave these issues for further research.

## DECLARATION OF INTEREST

None.

## Supporting information

Supplementary Material

## Footnotes

^1^
Also see the recent work of Jordà et al. (2020) on the long‐run consequences of historical pandemics dating back to the 14th century.

^2^
Newspaper evidence from the 1918 pandemic in the United States, collected by Correia et al. (2020), shows that workers calling sick disrupted production in many manufacturing plants. Velde (2020) found that sizeable labour shortages affected the coal industry and caused drops of up to 20% in capacity utilization during the peak of the pandemic.

^3^
As Correia et al. (2020) noted, the 1918 type of NPIs never reached the point of shutting down non‐essential businesses. At most, staggered business hours were introduced to avoid crowding.

^4^
An issue that is seldom discussed is whether severe recessions may also have negative health outcomes in the long run. Almond and Mazumder (2011) showed that reduced food consumption during pregnancy (as proxied by fasting during Ramadan among the Muslim population) leads to higher adult disability rates. In this sense, economic distress among the poorest may affect their long‐term health outcomes.

^5^
Some non‐anthropogenic sources of nitrogen dioxide also exist. Soil burning of natural origins is one example. Estimates of purely anthropogenic emissions of nitrogen oxides (NO*_x_*) are only possible through modelling.

^6^
See for instance Calabrese et al. (2013) and Hawelka et al. (2014) for an example of cellphone data used to track individual mobility patterns at the urban and global level respectively.

^7^
Country‐specific illustrations and detailed data description can be found in Demirgüc‐Kunt et al. (2020a).

^8^
Some of the differences in electricity consumption could be explained by the warmer weather in February 2020 compared to the same month last year (NOAA 2020; https://www.ncdc.noaa.gov/sotc/global‐regions/202002).

^9^
Civic capital appears to have played a role in the enforcement of social distancing measures (Durante et al., 2021).

^10^
To approximate the heating demand, we use the number of hours during the day when the ambient temperature was below 18°C (65°F); cooling demand is approximated by the number of hours in a day when the ambient temperature exceeded 24°C (75°F).

^11^
Note that this specification treats school closures independently of whether a ban of public events was in place or not, and it treats a partial or targeted lockdown independently of whether a ban of public events or a school closure was in place or not. The partial correlations captured by *β* for the ban of public events, school closure and partial lockdown therefore correspond to the average way in which these NPIs were implemented in the sample. This is not the case for the partial correlation corresponding to the full, national lockdown, the most stringent NPI, which necessarily includes a ban of public events, school closure and any kind of partial or targeted lockdown.

^12^
Using the daily number of infected individuals per million does not change the main results.

^13^
The model was initially developed by a team at the University of Basel. The online tool providing the country‐specific scenarios is available at https://covid19‐scenarios.org/.

^14^
Results are similar if each NPI dummy is included on its own in different specifications. The coefficients associated with the national lockdown, the ban of public events and school closure are separately negative and statistically significant.

^15^
We also estimate the regressions where we use daily infection rates as an indicator for the pandemic's progression. These estimations produce similar results in terms of the impact of the NPIs on both electricity and NO_2_ emissions. The effects of the daily infection rates on the economic activity indicators are not significant.

^16^
The Stock–Yogo weak identification test using the Cragg–Donald *F* statistic is highly significant as is the standard *F* test of the first stage.

^17^
Unreported regressions (available upon request) show that NPIs are also associated with a significant decrease in NO_2_ measurements at the city level. The city readings are obtained from the ground stations and may not be representative of a country's total emissions, while the national data are obtained from satellite readings. Hence, the two are not comparable.

^18^
The partial correlation between excess mobility drop (defined as the difference between the predicted drop in mobility for a given level of de jure stringency) and the speed of implementation is positive and statistically significant. The partial correlation between excess mobility drop (defined as the difference between the predicted drop in mobility for a given level of de jure stringency) and the speed of implementation is positive and statistically significant. The partial correlation between excess mobility drop (defined as the difference between the predicted drop in mobility for a given level of de jure stringency) and the speed of implementation is positive and statistically significant.

^19^
The average value of the Governance Effectiveness indicator (see World Governance Indicators, 2019 update in www.govindicators.org) for the five countries that had the worst health outcomes in our sample (Belgium, France, Italy, Spain and the United Kingdom as measured by the mortality rate per million inhabitants as of 25 April 2020) is 1.08 as compared to an average of 0.64 for the remaining countries in the region. The indicator ranges from −2.5 (weak) to 2.5 (strong) government performance.

^20^
The number of foreign residents born in China as reported by Eurostat and national statistical institutes in 2018 or latest available year. The numbers only include those with a valid residence permit.
