# Supplementary material for: The sooner, the better: The economic impact of non‐pharmaceutical interventions during the early stage of the COVID‐19 pandemic
Source: Economics of Transition and Institutional Change. 2021 Aug 22;29(4):551–73. doi: 10.1111/ecot.12284 (PMC8653187; doi:10.1111/ecot.12284)
Supplement: Supplementary file 1 — Supplementary Material [file ECOT-29-551-s001.docx]

**Appendix 1. Definition of NPI implementation dates**

The main source for information on the implementation of NPIs is the data set compiled by Coronavirus Government Response Tracker of the Blavatnik School of Government at Oxford University (<https://www.bsg.ox.ac.uk/research/research-projects/coronavirus-government-response-tracker>). We use the version of the data set published on April 30, 2020. The following criteria were used to determine the implementation dates:

1. Cancelation of public events:

Indicator C3 takes a value of 2 (“require canceling”)

1. School closure:

Indicator C1 takes a value of 3 (“require the closing of all levels”) or 2 (“require closing some levels”) (and C1_Flag takes a value of 1 (“General”)

1. Partial lockdown:
2. Indicator C2 (“workplace restrictions”) takes a value of 2 (“require closing for some category of workers”) or 3 (“require closing all-but-essential workplaces”) and C2_Flag takes a value of 0 (“Targeted”)

*Or*

1. Indicator C7 (“restrictions on internal movements”) takes a value of 1 (“recommend movement restriction”), and C2 takes a value of 2 (“require closing for some category of workers”)
2. Full lockdown:
3. Indicator C2 (“workplace restrictions”) takes a value of 2 (“require closing for some category of workers”) or 3 (“require closing all-but-essential workplaces”) and C2_Flag takes a value of 1 (“General”)

*Or*

1. Indicator C7 (“restrictions on internal movements”) takes a value of 2 (“movement restricted”) and C2 takes a value of 2 (“require closing for some category of workers”)

For countries not included in the Oxford Government Response Tracker, we used alternative sources, including news reports for full and partial lockdown measures and the World Bank Education COVID-19 Dashboard (<https://www.worldbank.org/en/data/interactive/2020/03/24/world-bank-education-and-covid-19>) for school closures. Table A.1 indicates the date of NPI implementation for each country.

Table A.1: NPI implementation dates by country.

| **Country** | **Cancelation of public events** | **School closure** | **Partial lockdown** | **Full lockdown** |
| --- | --- | --- | --- | --- |
| Albania | 9-Mar-20 | 9-Mar-20 | 9-Mar-20 | 12-Mar-20 |
| Armenia | 24-Mar-20 | 13-Mar-20 |  | 24-Mar-20 |
| Austria | 11-Mar-20 | 16-Mar-20 |  | 16-Mar-20 |
| Azerbaijan | 14-Mar-20 | 3-Mar-20 | 22-Mar-20 | 31-Mar-20 |
| Belarus |  |  |  |  |
| Belgium | 14-Mar-20 | 14-Mar-20 |  | 14-Mar-20 |
| Bulgaria | 13-Mar-20 | 5-Mar-20 |  | 13-Mar-20 |
| Bosnia and Herzegovina | 11-Mar-20 | 17-Mar-20 |  | 17-Mar-20 |
| Croatia | 10-Mar-20 | 14-Mar-20 | 14-Mar-20 | 20-Mar-20 |
| Cyprus | 10-Mar-20 | 13-Mar-20 |  | 16-Mar-20 |
| Czech Republic | 11-Mar-20 | 11-Mar-20 |  | 14-Mar-20 |
| Denmark | 16-Mar-20 | 13-Mar-20 | 13-Mar-20 | 18-Mar-20 |
| Estonia | 12-Mar-20 | 16-Mar-20 |  | 25-Mar-20 |
| Finland | 16-Mar-20 | 16-Mar-20 |  | 16-Mar-20 |
| France | 13-Mar-20 | 16-Mar-20 |  | 17-Mar-20 |
| Georgia | 31-Mar-20 | 2-Mar-20 | 19-Mar-20 | 31-Mar-20 |
| Germany | 10-Mar-20 | 16-Mar-20 |  | 22-Mar-20 |
| Greece | 9-Mar-20 | 11-Mar-20 |  | 23-Mar-20 |
| Hungary | 11-Mar-20 | 11-Mar-20 | 16-Mar-20 | 28-Mar-20 |
| Iceland | 24-Mar-20 | 17-Mar-20 |  |  |
| Ireland | 12-Mar-20 | 12-Mar-20 |  | 27-Mar-20 |
| Italy | 23-Feb-20 | 4-Mar-20 | 8-Mar-20 | 10-Mar-20 |
| Kazakhstan | 12-Mar-20 | 16-Mar-20 | 19-Mar-20 | 30-Mar-20 |
| Kosovo | 24-Mar-20 | 12-Mar-20 |  | 24-Mar-20 |
| Kyrgyz Republic | 12-Mar-20 | 16-Mar-20 |  | 25-Mar-20 |
| Lithuania | 16-Mar-20 | 12-Mar-20 |  | 16-Mar-20 |
| Luxembourg | 13-Mar-20 | 16-Mar-20 |  | 16-Mar-20 |
| Latvia | 14-Mar-20 | 12-Mar-20 | 14-Mar-20 |  |
| Moldova | 10-Mar-20 | 11-Mar-20 |  | 24-Mar-20 |
| North Macedonia | 18-Mar-20 | 10-Mar-20 |  | 18-Mar-20 |
| Malta | 22-Mar-20 | 17-Mar-20 |  | 22-Mar-20 |
| Montenegro | 30-Mar-20 | 13-Mar-20 |  | 30-Mar-20 |
| Netherlands | 10-Mar-20 | 15-Mar-20 |  | 15-Mar-20 |
| Norway | 12-Mar-20 | 12-Mar-20 |  | 16-Mar-20 |
| Poland | 10-Mar-20 | 12-Mar-20 |  | 14-Mar-20 |
| Portugal | 12-Mar-20 | 13-Mar-20 | 19-Mar-20 | 9-Apr-20 |
| Romania | 21-Mar-20 | 11-Mar-20 |  | 21-Mar-20 |
| Russian Federation | 10-Mar-20 | 23-Mar-20 |  | 30-Mar-20 |
| Serbia | 15-Mar-20 | 16-Mar-20 | 18-Mar-20 | 21-Mar-20 |
| Slovak Republic | 10-Mar-20 | 16-Mar-20 | 12-Mar-20 | 16-Mar-20 |
| Slovenia | 20-Mar-20 | 16-Mar-20 | 12-Mar-20 | 20-Mar-20 |
| Spain | 10-Mar-20 | 14-Mar-20 |  | 14-Mar-20 |
| Sweden | 12-Mar-20 |  |  |  |
| Switzerland | 25-Feb-20 | 13-Mar-20 |  | 17-Mar-20 |
| Tajikistan |  |  |  |  |
| Turkey | 16-Mar-20 | 16-Mar-20 | 22-Mar-20 |  |
| Turkmenistan |  |  |  |  |
| United Kingdom | 21-Mar-20 | 21-Mar-20 |  | 21-Mar-20 |
| Ukraine | 17-Mar-20 | 12-Mar-20 |  | 17-Mar-20 |
| Uzbekistan | 24-Mar-20 | 16-Mar-20 |  | 24-Mar-20 |

**Appendix 2. Estimation of the short-term elasticity between NO_2_ emissions and economic activity: The case of China**

Vehicles, power plants, and industrial facilities that use fossil fuels as energy emit substantial amounts of nitrogen dioxide (NO_2_) as a byproduct of their activity. In this appendix section, we provide a simple calculation of the elasticity between such measurements and actual economic indicators recently published by China for the period January-February 2020. This elasticity can be understood as an ‘exchange rate’ between NO_2_ emissions and economic activity, which can then allow for a high-frequency estimate of economic activity. Note that the elasticity we intend to estimate is short-term – it does not relate to long-term trends between emissions and growth, where technological changes may play a more considerable role than short-term economic disruptions.

The COVID-19 outbreak in the city of Wuhan led the Chinese government to impose a lockdown in the city on January 23, 2020, later extended to most of Hubei province on the following day, the whole province being on lockdown by January 28. The lockdown was lifted for parts of Hubei on March 13 and for Wuhan on April 8. The lockdown of Hubei coincided with the Lunar New Year holiday, which the Chinese government decided to extend for ten days (instead of the regular seven days) up to February 2 for all the country, with normal work expected to resume by February 3. However, provincial authorities delayed the resumption of normal work to February 10 throughout most of China (except Hubei).

The economic disruption driven by the extension of the Lunar New Year holiday, the lockdown in Hubei, and the mobility restrictions imposed in light of the spread of COVID-19 is evident when looking at the emissions of NO_2_ in China. Figure A.1 plots the evolution of the average NO_2_ concentration in the air of the country^[[1]](#footnote-1)^ over two periods of 14 weeks centered around the Lunar New Year in 2019 (February 5) and 2020 (January 25). While in the lead up to the Lunar New Year the NO_2_ was similar or slightly lower in 2020 than in 2019, after that date the emissions rebounded in 2019 but fell to lower levels in 2020. In the three weeks that followed the Lunar New Year holiday week, the average concentration of NO_2_ in 2019 (4.87 x 10^15^ molecules/cm^2^) was almost at the same level as in the last week before the holiday (4.95 x 10^15^ molecules/cm^2^), while in 2020 it was at half its level (an average of 2.50 x 10^15^ molecules/cm^2^ vs. a pre-holiday week average of 5.12 x 10^15^ molecules/cm^2^). The levels of NO_2_ in 2020 only started increasing in the fourth week after the Lunar New Year holiday. Overall, in the three weeks before the Lunar New Year, the average concentration of NO_2_ in 2020 was 89 percent of the same value in 2019, while for the three weeks after the holiday, the average value in 2020 was 53 percent of the 2019 value.

Figure A.1 – Average NO_2_ concentration in China, 2019 and 2020


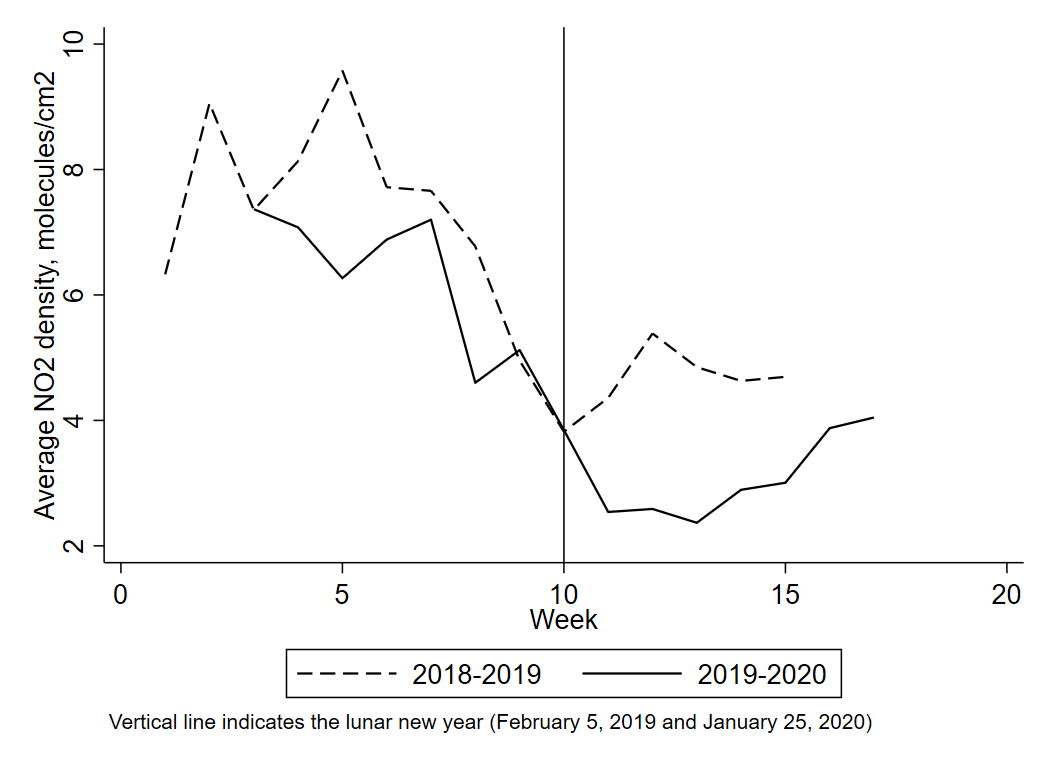


Note: this figure plots the value of average weekly NO_2_ density over Chinese territory (see footnote 21 before for precisions on this calculation) in December-February of years 2018-2019 and 2019-2020. The values corresponding to 2019-2020 have been shifted, so the lunar new year coincides with the year 2018-2019 (week 10 of 2019).

China’s National Bureau of Statistics has published consolidated economic indicators for the period January-February 2020. The value-added of industrial enterprises fell 13.5 percent on a year-to-year basis, while the index of services production dropped 13 percent in the same period. Total retail sales of consumer goods decreased by 20.5 percent when compared to the same two months of 2019. Separately, the Financial Times has created an index of daily economic activity for China,^[[2]](#footnote-2)^ showing that by mid-February 2020, economic activity was close to 50 percent below its value on January 1, 2020. The same index calculated for the equivalent period of 2019 was around 85 percent of the value of early 2020, suggesting a close to 40 percent year-to-year decrease in activity in mid-February 2020.

Table A.2 presents the calculation of a series of implied elasticities between NO_2_ concentration and economic activity based on different economic indicators. The benchmark against which NO_2_ concentration values in 2020 are compared corresponds to the average ratio between the three weeks before the Lunar New Year in 2020 and the same period in 2019 – this to take into account the fact that, for reasons beyond this analysis, the NO_2_ concentration in China had been slightly lower in 2020 than in 2019 even before any lockdown was imposed. Three economic indicators are expressed in bi-monthly terms, and therefore a comparable NO_2_ concentration is calculated over two months, while one indicator (FT China Economic Activity Index) is expressed in weekly terms. The implied elasticities range from 0.32 in the case of industrial value-added to 1 for the FT China Economic Activity Index. This suggests considerable variability in the relationship between NO_2_ concentration and economic activity. Despite this, the calculated elasticities allow estimating the potential economic consequences of lockdown-induced decreases in NO_2_ concentration.

A cleaner exercise would have relied exclusively on data from areas where a strict lockdown was imposed –like the province of Hubei– in order to ensure that observed variations in emissions are driven by the same factors as those explaining the variations in economic indicators. While NO_2_ concentration data are available for that province, no economic indicator has been yet published at that level, therefore making it impossible to estimate the corresponding elasticity. The elasticities calculated in table A.2 could, therefore, be biased by the presence of non-lockdown-induced variations in NO_2_ concentration, but, given the widespread economic disruption beyond the province of Hubei, this bias can be thought of as being small in relative magnitude.

Table A.2 – Calculation of elasticities between NO_2_ concentration and economic activity

| Average NO_2_ concentration | | | | Economic activity | | Implied elasticity |
| --- | --- | --- | --- | --- | --- | --- |
| Benchmark: 3 weeks pre-lunar new year, the ratio of 2020 vs. 2019 | Period | Ratio vs. 2019 | Difference with benchmark | Indicator | Difference with 2019 |  |
| (1) | (2) | (3) | (4) | (5) | (6) | (7) |
| 0.89 | January-February 2020 | 0.62 | -31% | Value added in industrial enterprises | -13% | 0.32 |
|  |  |  |  | Index of services production | -13.5% | 0.33 |
|  |  |  |  | Retail sales of consumer goods | -20.5% | 0.50 |
|  | 3 weeks post lunar new year | 0.53 | -41% | FT China economic activity index (mid Feb) | -40% | 1.00 |

Note: this table presents the calculation of elasticities between NO_2_ density over Chinese territory (columns 1 to 4) and short-term economic indicators (columns 5 and 6). The elasticities in column 7 are calculated as the ratio of the values in column 6 to the corresponding values in column 4.

1. Given the very low population density of China’s western half, only 50% of the surface of the country is used for this calculation. Data pixels (0.25 degree x 0.25 degree) for the whole Chinese territory are ordered based on their mean NO_2_ concentration in the 14 weeks of 2019 under consideration and the 50% with lowest values are dropped for the analysis. [↑](#footnote-ref-1)
2. See <https://www.ft.com/content/0c13755a-6867-11ea-800d-da70cff6e4d3> Data only available by visual inspection. [↑](#footnote-ref-2)
